# Lessons from the Titan submersible: A wake‐up call for radiation oncology

**DOI:** 10.1002/acm2.14121

**Published:** 2023-08-10

**Authors:** Mohammad Bakhtiari

**Affiliations:** ^1^ WellSpan Health Department of Radiation Oncology Chambersburg Pennsylvania USA

1

The world still grapples with the aftermath of the shocking implosion of the Titan submersible and its five occupants, an event that underscores the lethal consequences of sidelining safety concerns, minimizing cognitive diversity, and normalizing deviant practices. This incident should serve as a wake‐up call for leaders across sectors, especially those in Radiation Oncology as the field is considered a high risk domain, to take stock of their existing operational systems and adopt a more proactive approach to safety.

One of the crucial lessons from the Titan submersible tragedy lies in understanding the 'normalization of deviance' phenomenon,^1^ where unsafe or irregular practices become normalized over time due to their frequent occurrence without immediate detrimental consequences. What makes this particularly dangerous is its insidious nature; the deviation becomes the new norm so subtly that the associated risks might go unnoticed until a catastrophic event occurs. One of the most striking examples of this is the Space Shuttle Challenger disaster in 1986.

Similarly, within healthcare settings, normalization of deviance is alarmingly prevalent. Staff might bypass safety protocols due to time constraints, resource limitations, or a false sense of security based on previous 'successful' outcomes. Over time, these deviations from standard protocols can become the norm, setting the stage for potential catastrophes.

If leaders dismiss deviations because “nothing has happened so far,” or “it works just fine in other places,” they are inadvertently promoting a culture that tolerates, and even encourages, unsafe practices.

The company responsible for the Titan, fired an employee voicing concerns about the submersible's safety.^2^ This is indicative of a suppressive culture that discourages dissenting opinions and downplays safety concerns. This resonates eerily with situations in healthcare where safety concerns raised by frontline staff are often dismissed or not given the attention they deserve.

The tragic story of the Titan brings to the forefront the perils of ignoring cognitive diversity, seen when OceanGate chose to overlook the safety reservations expressed by a group of thirty‐eight experts with a wealth of different perspectives. In healthcare too, cognitive diversity is invaluable; the varied insights and perspectives can result in innovative solutions, better decision‐making, and improved patient outcomes.

The Titan incident serves as a stark reminder of the potential catastrophes that can ensue when leaders normalize deviance, suppress dissenting voices, and adhere rigidly to top‐down decision‐making. Let us commit to fostering a culture that prioritizes safety at all costs, respects cognitive diversity, and values transparent and collaborative decision‐making.

## AUTHOR CONTRIBUTIONS

Mohammad Bakhtiari: Author and corresponding author.

## CONFLICT OF INTEREST STATEMENT

The author declares no conflicts of interest.
